# Supplementary material for: Defect Engineering Enhances the Charge Separation of CeO2 Nanorods toward Photocatalytic Methyl Blue Oxidation
Source: Nanomaterials (Basel). 2020 Nov 21;10(11):2307. doi: 10.3390/nano10112307 (PMC7700514; doi:10.3390/nano10112307)
Supplement: Supplementary file 1 [file nanomaterials-10-02307-s001.pdf]

nanomaterials

Supplementary Materials

Defect Engineering Enhances the Charge Separation of CeO_2 Nanorods toward Photocatalytic Methyl Blue Oxidation

Jindong Yang [1], Ning Xie [2], Jingnan Zhang [1], Wenjie Fan [3], Yongchao Huang [2,*] and Yexiang Tong [1,*]

[1] MOE Laboratory of Bioinorganic and Synthetic Chemistry the Key Lab of Low-Carbon Chemistry and Energy Conservation of Guangdong Province, School of Chemistry, Sun Yat-sen University, Guangzhou 510006, China; usayjd@hotmail.com (J.Y.); zhangjn28@mail2.sysu.edu.cn (J.Z.)

[2] Institute of Environmental Research at Greater Bay Area, Key Laboratory for Water Quality and Conservation of the Pearl River Delta, Ministry of Education; Guangzhou Key Laboratory for Clean Energy and Materials, Guangzhou University, Guangzhou 510006, China; xiening0101@163.com

[3] Analysis and Testing Center, South China Normal University, Guangzhou 510006, China; 20175033@m.scnu.edu.cn

* Correspondence: huangych@gzhu.edu.cn (Y.H.), chedhx@mail.sysu.edu.cn (Y.T.)

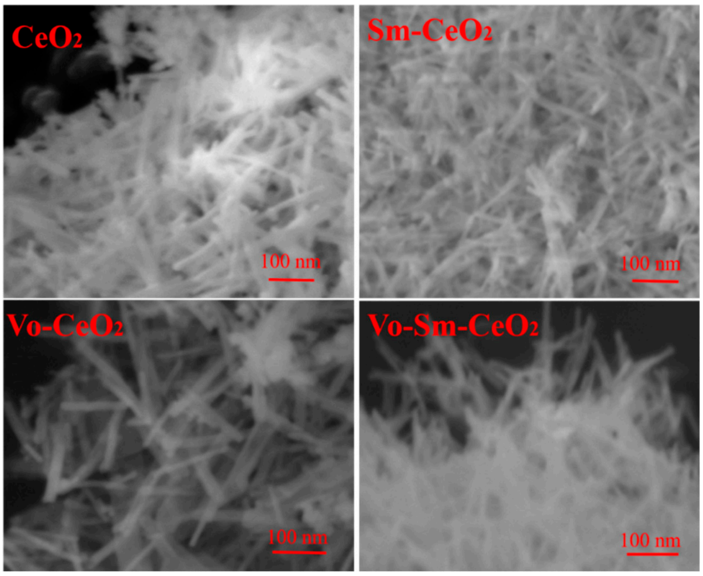

Figure S1. SEM images of CeO_2, Sm-CeO_2, Vo-CeO_2 and Vo-Sm-CeO_2.

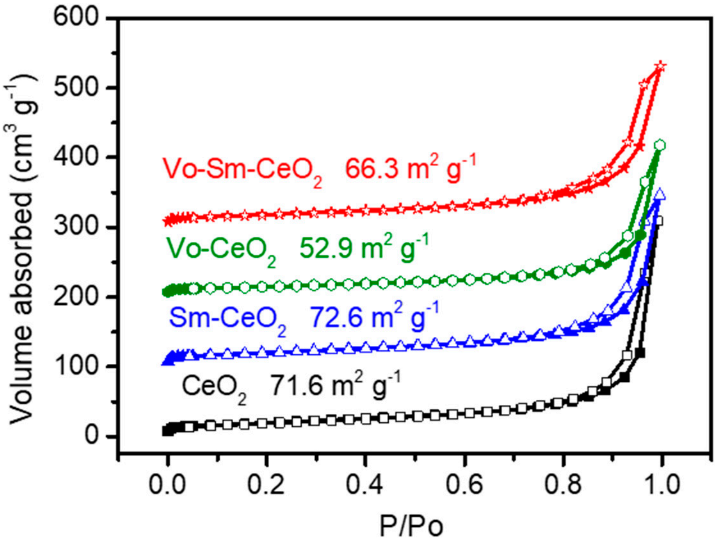

Figure S2. The N_2 adsorption spectra of all the samples.

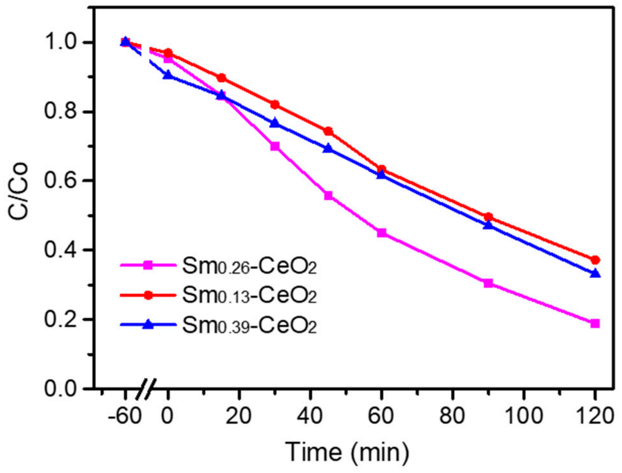

Figure S3. Photocatalytic performance of Sm doping CeO_2.

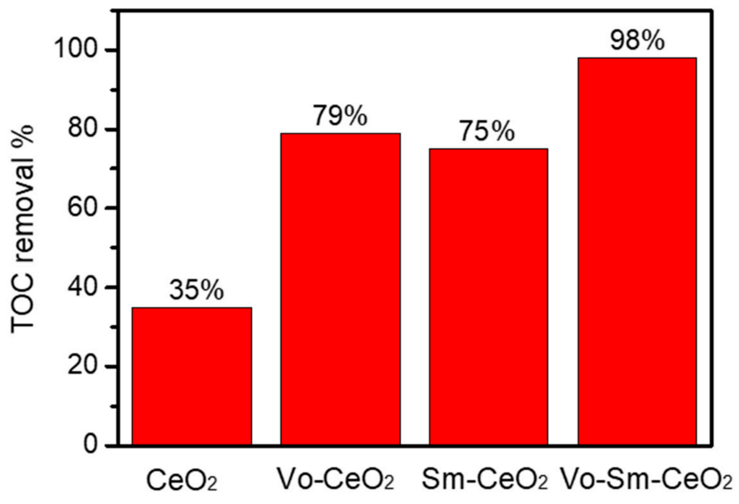

Figure S4. TOC removal efficiency CeO_2, Sm-CeO_2, Vo-CeO_2 and Vo-Sm-CeO_2 at 120 min.

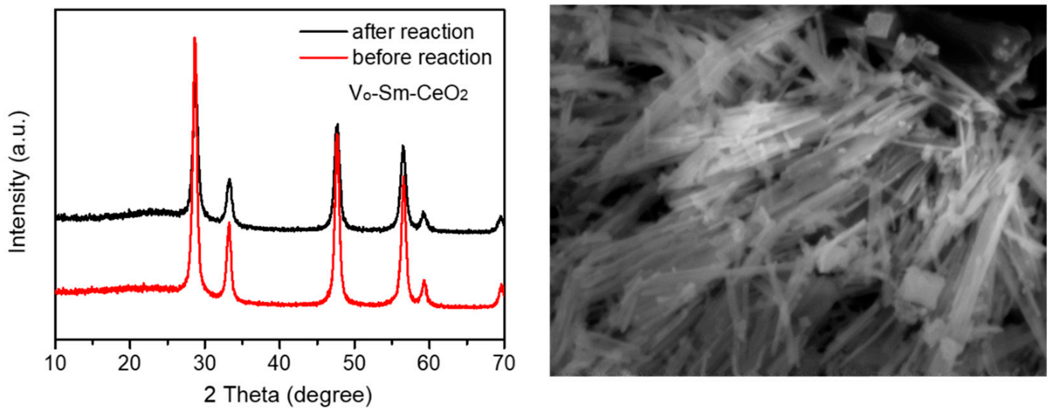

Figure S5. XRD spectra and SEM image of Vo-Sm-CeO_2 after five cycles test.

Table S1. The performance of Vo-Sm-CeO_2 is also compared with other reported Ce based photocatalysts.

Samples	Solution	Light Source	Photocatalytic Efficiency	Ref.
Vo-Sm-CeO_2 (0.02 g)	100 mL MB (10 mg/L)	300 W Xe lamp coupled with a 420 nm cut-off filter	*T50=30 min *T100=90 min	Our sample
$Ce_xCo_{1-x}O_2$ nanoparticle (0.01 g)	200 mL MB (10 ppm)	150 W Xenon lamp	T_{29}= 180 min	1
ZnO/CeO_2/10 wt.% NGP (0.03 g)	100 mL MB (20 mg/L)	150 W Xenon lamp	T_{84}=120 min	2
$CeCa_5$ quantum dots (0.2 g)	100 mL MB (5×10^{-3}M)	150 W Xenon lamp	T_{84}=50 min	3
CeO_2 Nps (0.1g)	100 mL MB (10 ppm)	125 W UV-light	T94=180 min	4
AgNWs@Ce O_2-2 (0.05g)	100 mL MB (10 ppm)	300 W Xe lamp	T_{80}=150 min	5
ZnO/CeO_2 (90:10) (0.5g)	500 mL MB	250 W projection lamp	T_{97}=150 min	6
CeO_2/Tb_2O_3 nanotubes (0.01g)	15 mL MB (0.3 mM)	50W Xenon lamp	T_{93}=75 min	7
5 wt% Zn-CeO_2 (0.5g)	500 mL MB (3×10^{-5} mol/L)	400 W sodium lamp	T_{85}=180 min	8
CeO_2/g-C_3N_4 (13.0%) (0.1g)	100 mL MB (10 mg/L)	300 W Xe lamp with a 400 nm cutoff filter	T_{95}=120 min	9
RGO/CeO_2 (0.015g)	100 mL MB (1×10^{-5} M)	direct sunlight	T_{50}=90 min	10
CeO_2 nanoparticles (0.05g)	100 mL MB (12ppm)	20 W UV light	T_{94}=27 min	11
Gd-CeO_2 nanoparticles (0.1g)	100 mL MB (1.7×10^{-6} M)	900W UV-light source	T_{98}=300min	12

*T50 means the time needs for photocatalytic degradation of 50% MB.

Reference

1. George S E, George M, Alex J, et al. Nonlinear optical and photocatalytic dye degradation of Co doped CeO_2 nanostructures synthesized through a modified combustion technique. Ceramics International, 2020.
2. Tju H, Shabrany H, Taufik A, et al. Degradation of methylene blue (MB) using ZnO/CeO_2/nanographene platelets (NGP) photocatalyst: Effect of various concentration of NGP[C]//AIP Conference Proceedings. AIP Publishing LLC, 2017, 1862(1): 030037.
3. Ramasamy V, Mohana V, Rajendran V. Characterization of Ca doped CeO_2 quantum dots and their applications in photocatalytic degradation. OpenNano, 2018, 3: 38-47.
4. Yadav L S R, Lingaraju K, Prasad B D, et al. Synthesis of CeO_2 nanoparticles: photocatalytic and antibacterial activities. The European Physical Journal Plus, 2017, 132(5): 239.
5. Wu L, Fang S, Ge L, et al. Facile synthesis of Ag@ CeO_2 core–shell plasmonic photocatalysts with enhanced visible-light photocatalytic performance. Journal of Hazardous Materials, 2015, 300: 93-103.

6. Rajendran S, Khan M M, Gracia F, et al. Ce3+-ion-induced visible-light photocatalytic degradation and electrochemical activity of ZnO/CeO_2 nanocomposite. Scientific Reports, 2016, 6: 31641.
7. Arul N S, Mangalaraj D, Kim T W. Photocatalytic degradation mechanisms of CeO_2/Tb_2O_3 nanotubes. Applied Surface Science, 2015, 349: 459-464.
8. Khan M A M, Khan W, Ahamed M, et al. Microstructural properties and enhanced photocatalytic performance of Zn doped CeO_2 nanocrystals. Scientific reports, 2017, 7(1): 1-11.
9. Huang L, Li Y, Xu H, et al. Synthesis and characterization of CeO_2/g-C_3N_4 composites with enhanced visible-light photocatatalytic activity. Rsc Advances, 2013, 3(44): 22269-22279.
10. Kaur J, Anand K, Anand K, et al. Reduced graphene oxide/CeO_2 nanocomposite with enhanced photocatalytic performance[C]//AIP Conference Proceedings. AIP Publishing LLC, 2015, 1661(1): 080009.
11. Tuyen L T T, Quang Khieu D, Long H T, et al. Monodisperse uniform CeO_2 nanoparticles: Controlled synthesis and photocatalytic property. Journal of Nanomaterials, 2016, 2016.
12. El Rouby W M A, Farghali A A, Hamdedein A. Microwave synthesis of pure and doped cerium (IV) oxide (CeO_2) nanoparticles for methylene blue degradation. Water Science and Technology, 2016, 74(10): 2325-2336.

© 2020 by the authors. Submitted for possible open access publication under the terms and conditions of the Creative Commons Attribution (CC BY) license (http://creativecommons.org/licenses/by/4.0/).
